# Phase 1 results of safety and tolerability in a rush oral immunotherapy protocol to multiple foods using Omalizumab

**DOI:** 10.1186/1710-1492-10-7

**Published:** 2014-02-20

**Authors:** Philippe Bégin, Tina Dominguez, Shruti P Wilson, Liane Bacal, Anjuli Mehrotra, Bethany Kausch, Anthony Trela, Morvarid Tavassoli, Elisabeth Hoyte, Gerri O’Riordan, Alanna Blakemore, Scott Seki, Robert G Hamilton, Kari C Nadeau

**Affiliations:** 1 Allergy, Immunology, and Rheumatology Division, Stanford University, 269 Campus Drive, CCSR3215c, Stanford, CA 94305, USA; 2 Dermatology, Allergy and Clinical Immunology Reference Laboratory, Johns Hopkins University School of Medicine, Baltimore, Maryland, USA

**Keywords:** Food allergy, Oral immunotherapy (OIT), Specific Oral Tolerance Induction (SOTI), Multiple food allergy, Safety, Efficacy, Omalizumab, Desensitization

## Abstract

**Background:**

Up to 30% of patients with food allergies have clinical reactivity to more than one food allergen. Although there is currently no cure, oral immunotherapy (OIT) is under investigation. Pilot data have shown that omalizumab may hasten the ability to tolerate over 4 g of food allergen protein.

**Objective:**

To evaluate the safety and dose tolerability of a Phase 1 Single Site OIT protocol using omalizumab to allow for a faster and safe desensitization to multiple foods simultaneously.

**Methods:**

Participants with multiple food allergies received OIT for up to 5 allergens simultaneously with omalizumab (rush mOIT). Omalizumab was administered for 8 weeks prior to and 8 weeks following the initiation of a rush mOIT schedule. Home reactions were recorded with diaries.

**Results:**

Twenty-five (25) participants were enrolled in the protocol (median age 7 years). For each included food, participants had failed an initial double-blind placebo-controlled food challenge at a protein dose of 100 mg or less. After pre-treatment with omalizumab, 19 participants tolerated all 6 steps of the initial escalation day (up to 1250 mg of combined food proteins), requiring minimal or no rescue therapy. The remaining 6 were started on their highest tolerated dose as their initial daily home doses. Participants reported 401 reactions per 7,530 home doses (5.3%) with a median of 3.2 reactions per 100 doses. Ninety-four percent (94%) of reactions were mild. There was one severe reaction. Participants reached their maintenance dose of 4,000 mg protein per allergen at a median of 18 weeks.

**Conclusion:**

These phase 1 data demonstrate that rush OIT to multiple foods with 16 weeks of treatment with omalizumab could allow for a fast desensitization in subjects with multiple food allergies. Phase 2 randomized controlled trials are needed to better define safety and efficacy parameters of multi OIT experimental treatments with and without omalizumab.

## Introduction

Up to 8% of the pediatric population suffers from food allergy and of those 30% have clinical reactivity to more than one food allergen [1-3]. The estimated cost of food allergies in the U.S. every year is approximately 25 billion U.S. dollars, with most of the burden (~$20 billion) borne by families themselves due to time lost from work, changing careers and emergency room visits [4]. Compared to those with single food allergies, multi-sensitized subjects experience a greater decrease in quality of life [5], are more likely to suffer from dietary deficiencies [6] and are less prone to spontaneously outgrow their allergies [7].

Oral, sublingual, and epicutaneous allergen-specific immunotherapies have been proposed as possible methods of desensitization for foods. Several prior studies have shown some success in using these approaches for single specific food allergens such as milk [8-15], egg [13,14,16-18], peanut [19-24], and hazelnut [25]. These current types of experimental treatments need to be tested for optimization in safety, efficacy, and length of time [26-34]. Safety is of critical importance at all phases of any protocol (initial dose escalation day, dose escalation, and maintenance phases) and allergic reactions while on OIT remain an important feature in long-term follow-up studies and in determining the overall success of food allergen immunotherapy [35]. However, one major limitation to the clinical application of current protocols is their use in participants with more than one food allergy, which would require multiple sequential rounds of immunotherapy over many years. We have recently reported that up to 5 allergens can be desensitized simultaneously without an increase in reaction rate when compared to single allergen desensitization [36]. However this protocol remained time consuming with a median of 85 weeks to reach maintenance dose (range = 54–156).

The use of IgE immunomodulatory therapies, including monoclonal antibodies and small molecules, has been under investigation in food allergies and has been reviewed recently in the literature [37-45]. Specifically, omalizumab has been shown to increase the threshold for adverse reactions on food challenge by up to 80 fold [41]. After obtaining pharmacodynamic data using basophil assays and free IgE measurements in subjects with food allergies who received standard omalizumab dosing, we found that 8 weeks post standard omalizumab therapy is an optimal time to start oral immunotherapy [46,47]. This concept of rush immunotherapy with omalizumab was previously used in immunotherapy studies involving pollens, milk and peanut with promising results [28,30,48-52]. Combined with food OIT, omalizumab is posited to increase dose tolerability, thus allowing for the possibility of a higher initial starting dose and faster treatment progression.

The objective of this trial was to study the safety and dose tolerability of a phase 1, open-label, rush OIT protocol, which included up to 5 foods simultaneously. The primary endpoint of our investigation was safety (i.e. the occurrence of allergic reactions throughout the course of the study). The secondary endpoints (i.e. tolerability) were i) the time to reach and maintain doses of 300 mg, 1000 mg and 4000 mg per food allergen protein as well as ii) a 10 fold increase from the baseline reactivity threshold to each of the food allergen proteins.

## Methods

This open-label, phase 1 study was performed in a single center hospital setting, with Institutional Review Board (IRB) and Investigational New Drug (IND) approvals. This project was approved by the IRB committee at Stanford University.

### Participant selection

Participants were eligible for inclusion if they: (1) were older than or equal to 4 years old; had proven sensitivity to at least two food allergens documented by both (2) a skin prick test (with neat extracts from Greer Laboratories, Lenoir, NC) greater than 3 mm (wheal), and (3) food-specific IgE greater than 0.35 ku/L (ImmunoCAP); (4) had clinical reactivity to those food proven by positive allergic reaction in a double-blind placebo-controlled oral food challenge (DBPCFC) as described below; and (5) had signed informed consent. Specific food allergens that were eligible for inclusion in this trial included cow’s milk, egg, peanut, nuts, grains and sesame seed. Exclusion criteria included: (1) eosinophilic esophagitis; (2) autoimmune disease; (3) severe cardiac disease; chronic treatment with (4) beta-adrenergic antagonists or (5) steroids; (6) a history of severe anaphylaxis requiring admission to an intensive care unit; (7) frequent allergic or non-allergic urticaria; and (8) poorly controlled asthma (defined as FEV1 below 80 percent of predicted).

DBPCFCs were performed on different days and separated by 72 hours for each qualifying food allergen and for the placebo (oat, or rice flour if allergic to oat). All participants performed spirometry, as appropriate per age, and had continuous pulse-oximetry and vital sign monitoring, every 15 minutes prior to and following increasing doses of placebo or allergenic food protein. DBPCFC doses were increased over 3.5 hours up to a cumulative dose of 182 mg food protein until an objective reaction occurred. Clinical reactivity was based on Bock’s criteria (grade 1 or above) [53]. The DBPCFC procedure used was described in a previous publication [36].

DBPCFCs and dose escalations occurred in a hospital with immediate access to a trained physician and study personnel. Given that reactions are expected to occur with OIT, training for the use of and indication for auto-injectable epinephrine was given to all participants and families/guardians at screening, on the initial dose escalation day and every three months during OIT. Our method of epinephrine training was described in detail in a previously [36].

### Study medications

#### Food flours/powders

This study used food flours/powders dispensed through a Food Flour/Powder GMP facility at Stanford (as per FDA guidelines (http://www.fda.gov/downloads/Drugs/GuidanceComplianceRegulatoryInformation/Guidances/ucm070273.pdf). A *Chemistry and Manufacturing Control* (CMC) section for each food allergen powder/flour included assessments for stability, identity, relative sterility, and purity of each of the food powders/flours. The food flours/powders we used include milk powder (Organic Valley, WI), egg powder (Deb El, NJ), peanut flour (Byrd Mill, VA), walnut flour (Carriere Family Farms, CA), cashew flour (Digestive Wellness, NY), almond flour (Just Almonds, NV), pecan flour (Green Valley, AZ), hazelnut flour (Holmquish Hazelnut Orchards, WA), wheat flour (Gold Medal, MN), soy flour (Honeyville Grain, Inc., UT), and sesame seed flour (Dispasa USA, Inc., TX). Each dose was weighed out by a trained professional on a professional-grade balance. Flour/powder protein content was calculated according to nutritional information provided by manufacturers and confirmed through protein assays run at Stanford laboratories.

#### Omalizumab

Omalizumab (Genentech, CA) was prepared and administered according to the product insert. Doses were determined based on weight and total IgE levels as per Omalizumab Global Dosing schedule as outlined in the online supplement (Additional file 1: Table S1).

### Study design

#### Pre-treatment with omalizumab

After enrollment, participants (n = 25) were pre-treated with omalizumab for 8 weeks according to the product insert dosing schedule to equilibrate with anti-IgE mAb (Additional file 1: Table S1). Omalizumab injections were administered at the hospital’s Clinical Translational Food Unit (CTFU) under medical supervision and patients were observed for at least one hour after injection.

#### Initial escalation

**Figure 1:**
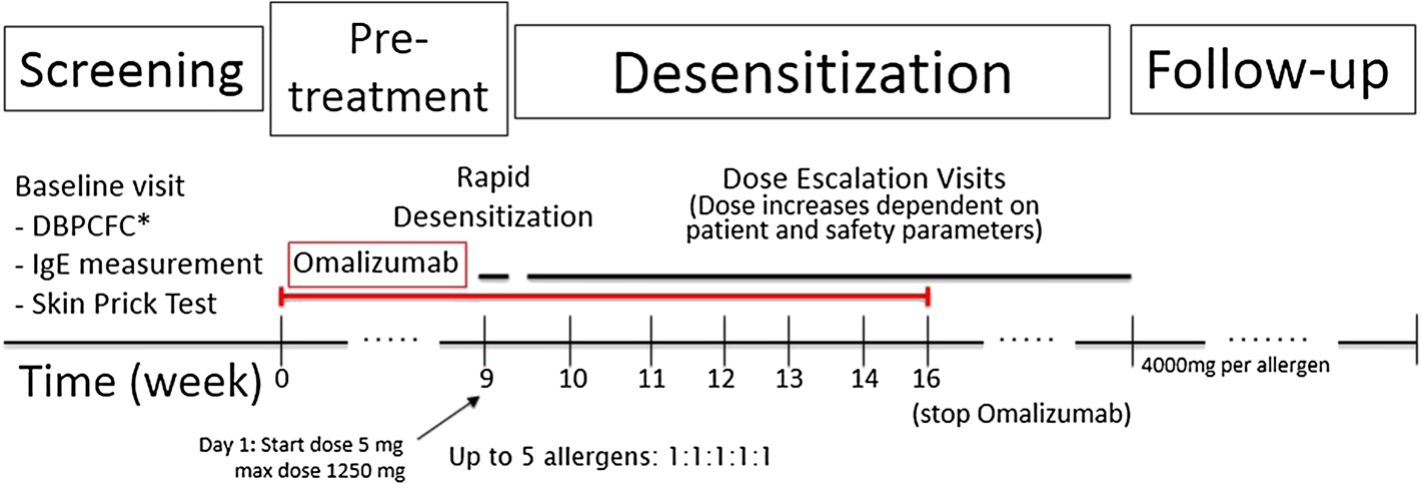
**Rush mOIT protocol timeline.** Amount of maintenance dose depends on number of allergens dosed (4000 mg per allergen). *Double-blind, placebo-controlled food challenges (DBPCFCs).

On the 9^th^ week after the 1^st^ injection of omalizumab, the participants underwent rapid oral desensitization to up to five offending food allergens (Figure 1). The OIT regimen was customized to what the participant was found to be allergic to in their baseline DBPCFCs. As many as 5 allergens (in 1:1:1:1:1 proportions) could be included in an OIT treatment plan, provided all selected allergens met the inclusion criteria.

All participants were admitted to the CTFU (clinical trial food unit) and vital signs (heart rate, respiratory rate, temperature, blood pressure and pulse oximetry) were monitored every 15 minutes. Trained clinical staff administered doses of food allergen. Antihistamines, inhaled beta-2 agonists, prednisolone, and injectable epinephrine were all readily available at the bedside.

On the initial escalation day, dosing began at 5 mg total food allergen protein divided equally between each of the offending food allergens (i.e. 1 mg protein of each allergen if 5 were given) and doses were slowly increased until the participant reached a final dose of 1250 mg protein (i.e. up to 250 mg protein of each offending food allergen if the participant’s regimen included 5 allergens). Food allergens were given over a period of 2.5 hours as outlined in Table 1. Participants were monitored for vital signs and physical assessments throughout the dosing process and were observed for an additional 2 hours after receiving the final dose. The highest tolerated dose (i.e. with no clinical reactivity) determined the participant’s starting daily home dose (up to a total dose of 1250 mg protein, divided evenly into each of the separate offending food allergens).

**Table 1:** Rush mOIT initial escalation day schedule

**Dose in mg of protein**	**Dosing interval in minutes**
5	30
50	30
150	30
300	30
625	30
1250	120

#### Home dosing

Individual doses were provided containing all of the participant’s allergens. Participants were instructed to ingest their dose after a full meal at approximately the same time each day. Each food allergen was given simultaneously in applesauce or pudding (or another medium the participant had shown tolerance to during placebo challenge). They were instructed not to miss their daily dose. Participants and their families were given instructions on how to monitor for reactions at home and record any symptoms in their dosing diary. Research staff kept in close contact with participants and families to investigate and document any adverse events. All families and participants had 24-hour contact information for study personnel in case of an allergic reaction and/or questions at any point during the study. All participants were provided with injectable epinephrine devices, oral antihistamines, and a treatment plan for possible allergic reactions. They were trained on the use of self-injectable epinephrine. Participants were instructed to avoid physical exertion 1 hour before and 2 hours after dosing and to contact the on-call service in the event of infection or environmental allergies. The goal of the OIT was to achieve a daily maintenance dose of 4000 mg protein of each allergen (up to 20,000 mg protein cumulative dose for participants taking 5 allergens in their OIT).

#### Dose escalation

The participants returned to the CTFU every two weeks for a dose escalation visit with daily home diaries, which detailed any symptoms that occurred and treatments given during the daily home dosing for the prior 2 weeks. Staff reviewed the dose diaries with the participants and their families at each visit. If home daily protein flour/powder doses had been well tolerated, the dose was increased in the hospital setting according to a predetermined scale as outlined in Table 2. Trained clinicians in the CTFU monitored participants for at least one hour following their new dose. If the new dose was tolerated, it subsequently became their daily dose for the following two weeks; otherwise they continued on their previous dose. Importantly, OIT protocols did not advance according to a fixed calendar, but, rather were individualized according to participants’ allergic reactions and safety outcomes.

**Table 2:** Rush mOIT dose escalation schedule

**Dose of protein (mg)**	**Interval in weeks**	**% of increase from previous**
2350 mg	2	88%
4000 mg	2	70%
5800 mg	2	45%
7600 mg	2	50%
9400 mg	2	30%
11200 mg	2	20%
14000 mg	2	25%
17500 mg	2	25%
20000 mg	2	14%

#### Omalizumab discontinuation

Omalizumab treatment was discontinued 8 weeks after the initial escalation day of food allergens, totaling 16 weeks total of omalizumab treatment.

#### Allergy testing

Peanut was the most frequent food allergen determined in the 25 participants (Additional file 1: Tables S2). Specific skin prick test (peanut extract from Greer Laboratories, Lenoir, NC) and serologies were compared at baseline and after a year of therapy. Sera were analyzed for peanut-specific IgE and IgG4 levels at John Hopkins Allergy and Clinical Immunology Reference Laboratory by immunoCAP FEIA (Thermofisher Scientific/Phadia, Kalmazoo, MI). IgE antibody levels < 0.1 kU_A_/L and IgG4 antibody levels <0.01 kU_A_/L were considered undetectable.

#### Statistical analysis

Dose progression was measured as the time to reach: 1) a 10-fold increase from initial cumulative dose eliciting a reaction on DBPCFC to each food allergen; as well as doses of 2) 300 mg; 3) 1000 mg; and 4) 4000 mg protein per food allergen. Food allergy testing results before and after therapy were compared with the Wilcoxon paired T test. All analyses were performed using GraphPad PRISM software version 6.0b (GraphPad, LaJolla, CA).

## Results

### Overall

Of a total of 53 participants screened, 25 met inclusion criteria and were enrolled in the phase 1 protocol. Detailed food allergen diagnoses are available in the online supplement (Additional file 1: Tables S3). Initial baseline clinical characteristics and number of allergens are presented in Table 3.

**Table 3:** Subjects baseline characteristics

**Number of subjects**	25
**Median age in yrs (range)**	7.4 (4.5-15.4)
**Male**	19 (76%)
**Clinical reaction**	
Respiratory	5 (20%)
Gastro-intestinal	13 (52%)
Epinephrine	1 (4%)
**Number of foods dosed**	
2	7 (28%)
3	4 (16%)
4	7 (28%)
5	7 (28%)
**Peanut baseline allergy test (if included in mix) (median and range)**	
SPT in mm	13 (3.5-26)
Specific IgE in kUa/L	31 (1–192)
Lowest amount triggering reaction in DBPCFC in mg protein	15.5 (1.6-100)
**Highest baseline allergy test (median and range)**	
SPT in mm	17 (6–29.5)
Specific IgE in kUa/L	66 (2–256)
Lowest amount triggering reaction in DBPCFC in mg protein	6 (0.1-100)
**Total IgE in kuA/L (median and range)**	645 (67–1829)

Over the study period, there were 3 withdrawals because of non-compliance with study medication. Overall, a total of 227 hospital-based dose escalation doses and 7,530 home doses were given (Table 4). Throughout the study, no participant missed more than 3 doses consecutively as recorded per their dose diaries.

**Table 4:** Reaction rates with rush mOIT

**Initial escalation day**	
*Escalations performed*	25
*Reactions*	13 (52%)
*Mild (Grade 1)*	13 (52%)
*Moderate (Grade 2)*	0
*Severe (Grade 3)*	0
*Epinephrine use*	0
**Dose escalations**	
*Doses administered*	227
*Reactions*	13 (5.7%)
*Mild (Grade 1)*	13 (5.7%)
*Moderate (Grade 2)*	0
*Severe (Grade 3)*	0
*Median reaction rate [range]*	0% [0–25]
*Epinephrine use*	0
**Home dosing**	
*Doses administered*	7530
*Reactions*	401 (5.3%)
*Mild (Grade 1)*	385 (5.1%)
*Moderate (Grade 2)*	15 (0.2%)
*Severe (Grade 3)*	1 (0.01%)
*Median reaction rate [range]*	3.2% [0.1-18.5]
*Epinephrine use*	1 (0.01%)

### Safety

**Figure 2:**
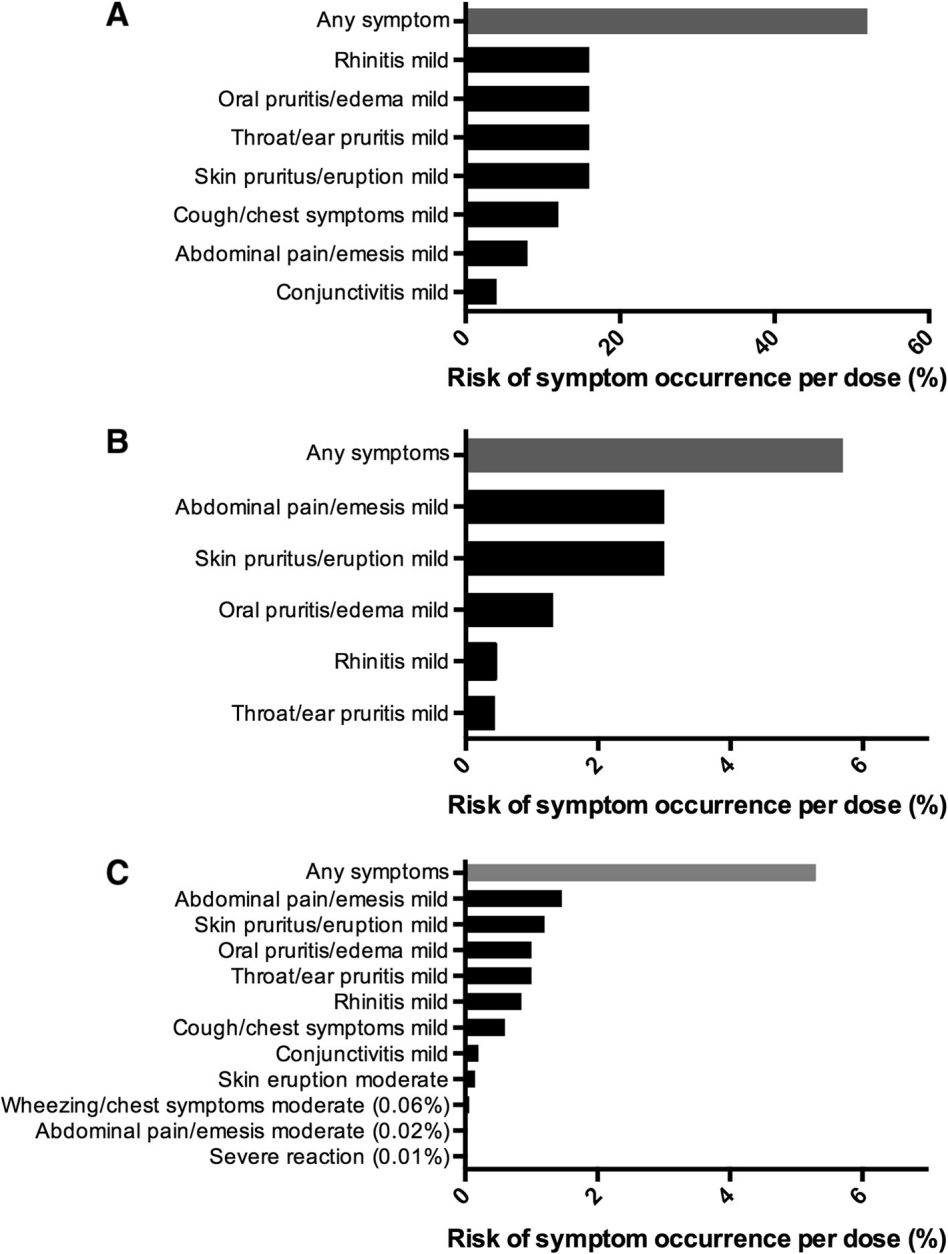
Symptom occurrence during rush mOIT with (A) initial escalation day, (B) dose escalations and (C) home dosing.

**Figure 3:**
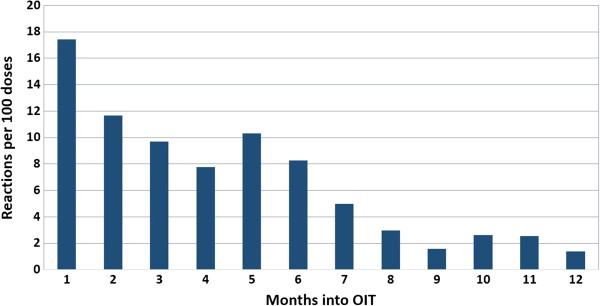
Time distribution of home dosing reactions for the first year of rush mOIT.

Rates and nature of dose reactions are depicted in Figure 2 for initial dose escalation day, dose escalations, and home dosing. Most (94%) allergic reactions were mild and included mainly abdominal pain, pruritus, and local ENT symptoms. Although 13 participants (52%) experienced some symptom on their initial dose escalation day, 19 (76%) were able to reach the full 1250 mg of food protein total. With home dosing, 401 of the 7530 doses (5.3%) triggered reactions with a median reaction rate of 3.2 per 100 doses [0.1-18.5]. Most home reactions occurred in the first months of therapy, with reaction rates dropping by 70% after 6 months of therapy, from 11 to 3 reactions per 100 doses (p < 0.0001) (Figure 3).

Throughout the trial, there were no serious adverse events. One severe reaction occurred shortly after reaching maintenance phase (16,000 mg) in a participant desensitized to peanut, almond, milk and egg. He presented with wheezing, abdominal pain and throat tightness 30 minutes after his dose, which resolved 5 minutes after the self-injection of epinephrine. The family did not report any obvious triggers such as exercise or viral infection.

### Dose progression

**Figure 4:**
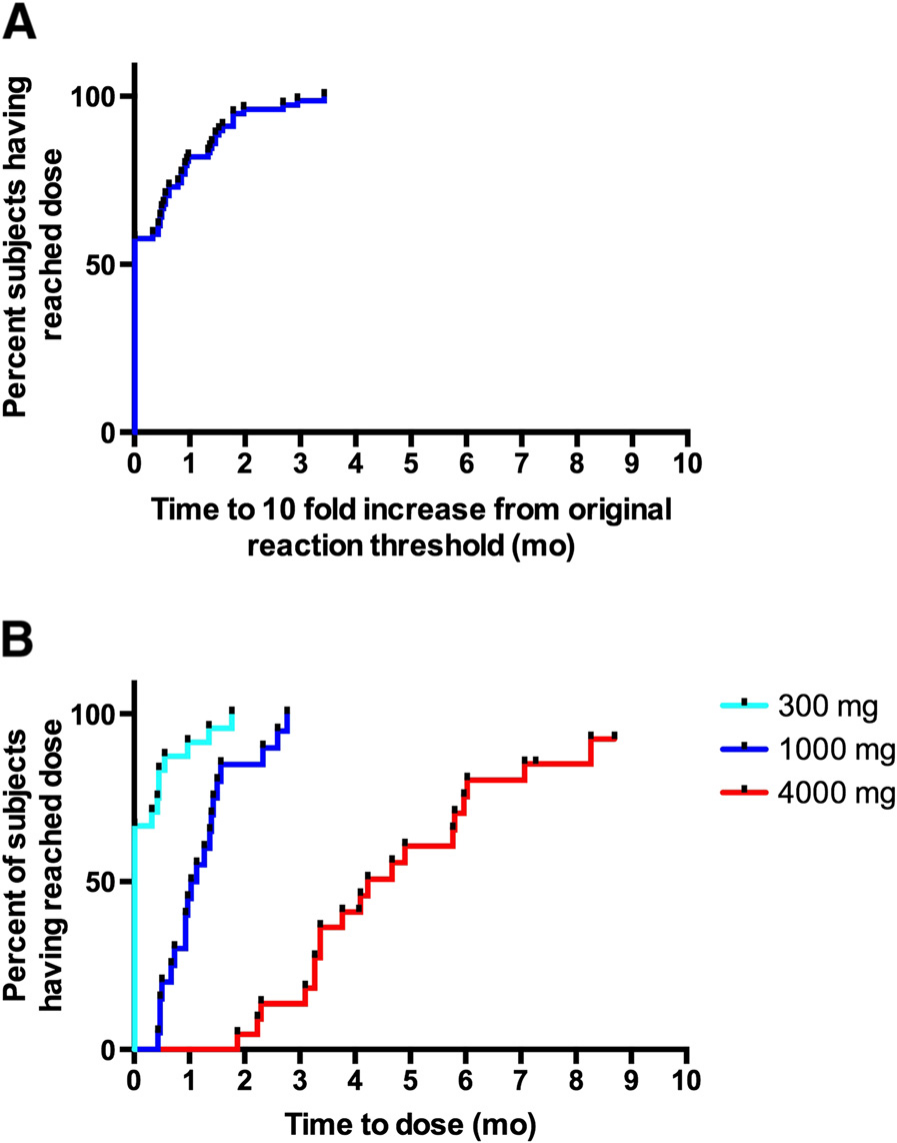
Kaplan-Meier curves showing (A) time to reach the dose corresponding to a 10 fold increase from the threshold at which the patient reacted to index foods on initial DBPCFC (each food reported as a separate event) as well as (B) time to dose of 300 mg, 1000 mg, and 4000 mg protein per each allergen.

As up-dosing was dependent on tolerance to the current dose, dose progression was treated as a marker of tolerability. Kaplan-Meier curves showing time to reach and maintain a 10-fold increase in threshold dose of index food allergen protein, as well as time to reach a dose of 300 mg, 1000 mg, and 4000 mg per food allergen protein are presented in Figure 4. The median time to reach maintenance dose (4000 mg per allergen) was 18 weeks [7–36 weeks] with all participants able to reach this dose by 9 months. All participants had reached a dose equivalent to a 10-fold increase of all their allergens by 2 months of therapy.

### Allergy testing

**Figure 5:**
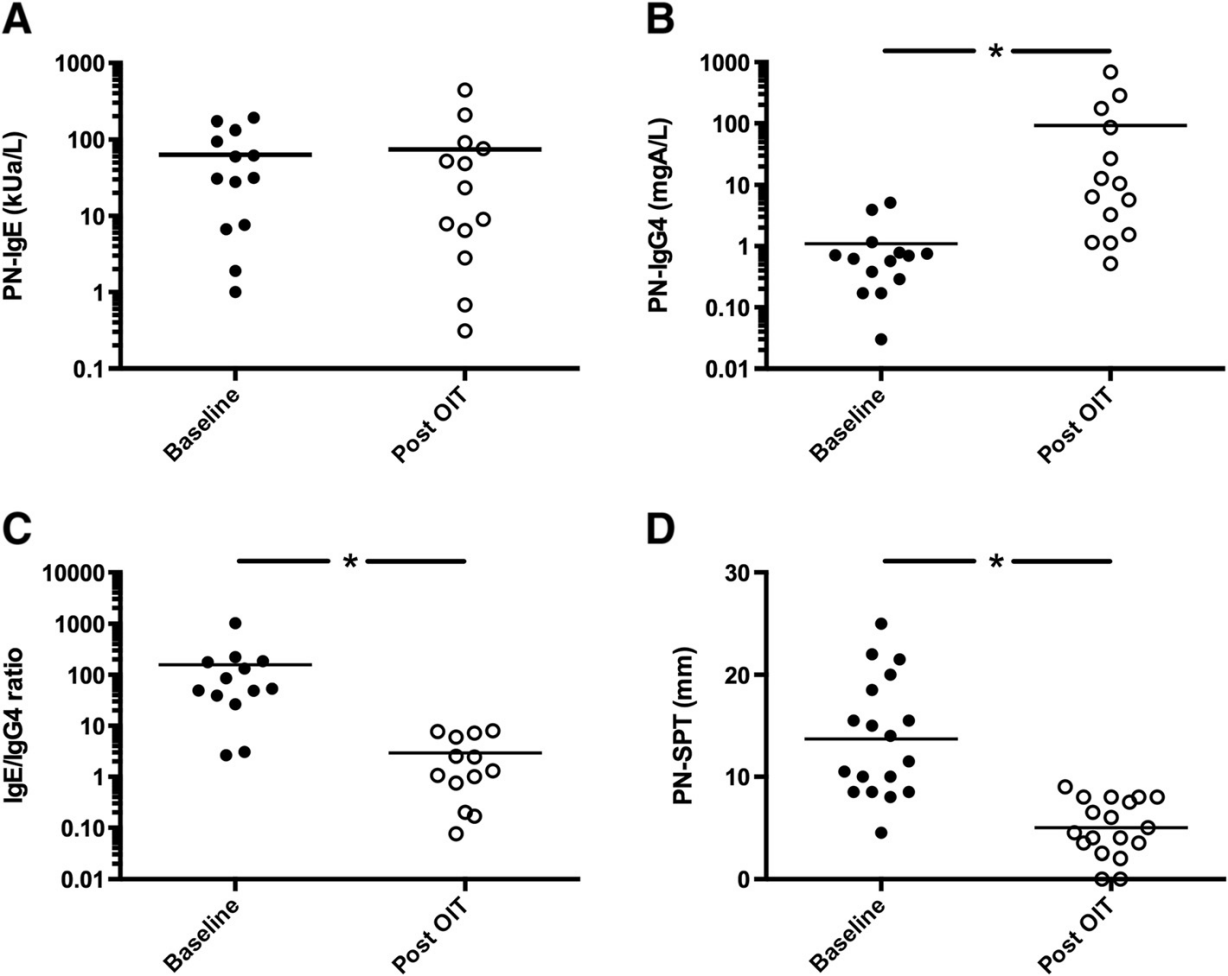
Peanut specific (A) IgE, (B) IgG4, (C) IgE/IgG4 ratio and (D) skin prick test results at baseline and after a year of therapy for participants with proven peanut allergy (* indicates p < 0.0001).

After 52 weeks of therapy, peanut-specific IgE (PN-IgE) did not change significantly (Figure 5). However, peanut-specific IgG4 (PN-IgG4) levels showed median increases of 8.23 mgA/L (p < 0.0001) while peanut SPT decreased by a median of 8 mm (p < 0.0001).

## Discussion

In this phase 1 safety study, we have shown that participants allergic to multiple foods were safely and rapidly desensitized to up to five food allergens simultaneously, using a rush OIT protocol with concomitant treatment with omalizumab. To the best of our knowledge, this is the first study to use omalizumab with OIT to multiple allergens simultaneously. These findings are particularly relevant considering the already high (~30%) and likely growing number of food allergic participants who are sensitized to more than one food allergen [3,53-56].

This study was designed as a proof of concept, open-label phase 1 study, with safety measurements as the primary endpoint. The rate of reactions observed in the rush mOIT group was similar to a group with the same eligibility and demographics undergoing mOIT in a previous study without omalizumab, despite the more rapid desensitization schedule [36]. The goal of adding omalizumab in this phase 1 study was primarily to enable rapid desensitization rather than to suppress allergic symptoms during OIT.

As the half-life of omalizumab is 24 days, we further hypothesized that any protective safety effect might wane over time. Participants were observed closely for the development of symptoms, including hives, worsening of eczema, or wheezing after omalizumab discontinuation (at 8 weeks after initial dose escalation), and were instructed to keep a diary of food allergy symptoms throughout the study. Our data show that the home reaction rate actually went down after 24 weeks of therapy from 11 to 3 per 100 doses (p < 0.0001) (Figure 4). This increase in safety could relate to the fact that participants were not up-dosing anymore at that point. However, the only use of epinephrine occurred shortly after the participant had reached the maintenance phase, thus vigilance should not be relaxed at any point. Rescue epinephrine was also required during the maintenance phase of previous rush studies using omalizumab (2 of 2 and 1 of 4 in peanut and milk rush OIT respectively) [28,52].

In addition to the safety data, this phase 1 study of rush mOIT provides initial preliminary evidence of increased dose tolerability. The median time at which participants on rush mOIT reached their maintenance dose (4000 mg per allergen) was 67 weeks earlier than that reported in a previous report on mOIT without omalizumab [36]. This represents a difference of about 34 dose escalations and about 67 additional weeks of enrollment. This might be relevant from a pharmaco-economic perspective. Considering a cost per visit in 2013 of approximately $160 [approximate cost of an office open food challenge per MediCare or public health insurance in Canada (RAMQ)], those 34 extra visits represent a minimal additional cost of approximately $5,440 in 2013 [57]. This could possibly offset the cost of omalizumab at the current time, which varies between $2,164 and $10,824 for 16 weeks, depending on the patient’s weight and total IgE levels. Furthermore, these calculations do not take into account the additional cost and impact of absenteeism from school and work for the participant and his/her parents during these approximate 34 additional visits [4]. However, one should be cautious when comparing these two phase 1 trials as the dose progression schedules were different. A phase 2 study comparing omalizumab to placebo in participants with a similar dosing schedule is needed to truly assess the efficacy gained from the addition of omalizumab to mOIT.

There are limitations to this study. Oral immunotherapy regimens were customized to the participant’s food allergies. This has led to some diversity when comparing the composition of specific food allergies between subjects. However, no one food allergen was found to be associated with greater dose tolerability or safety. This is consistent with one of the key long term goals of the study which was to begin to develop customized, patient-based, regimens for oral immunotherapy that could be tested for safety, and dose tolerability.

Importantly, our study showed desensitization but not tolerance. Clinical tolerance is proven by demonstrating sustained unresponsiveness to the food after stopping the maintenance dose for a prolonged period of time. Future phase 2 trials on the use of omalizumab combined with OIT will be useful to see if omalizumab affects this outcome.

Our cohort did not include subjects with high total serum IgE levels as this is sometimes the case for children with multiple food allergies. Three subjects had total serum IgE slightly greater than 1500 kUa/L and received the maximum dose of Omalizumab (600 mg every 2 weeks). The optimal dosing for subjects with higher levels would require further study.

Serological analyses were performed for peanut to allow for consistent comparisons between participants, as this was the most frequent allergen. The serologic changes after 52 weeks of therapy were identical to those previously reported in subjects undergoing non-rush OIT (without omalizumab) [36,58].

In conclusion, the data from a single site, phase 1 study demonstrate that a rush OIT protocol to multiple food allergens using adjunct omalizumab can be performed safely in a hospital setting. At this time, rush mOIT is an experimental treatment and should be conducted by trained research personnel with immediate access to emergency equipment. Phase 2, blinded, multicenter trials are needed to continue to determine safety and efficacy parameters of rush mOIT in larger numbers of multi-sensitized participants.

## Abbreviations

CTFU: Clinical trial food unit; DBPCFC: Double-blind placebo-controlled food challenge; EMEA: European Medicines Agency; FDA: Food and drug administration; GMP: Good manufacturing practice; IND: Investigational new drug; IRB: Institutional review board; mOIT: Multiple allergen oral immunotherapy; PN-IgE: Peanut specific Immunoglobulin E; PN-IgG4: Peanut specific immunoglobulin G4; SPT: Skin prick test.

## Competing interests

The authors have no relevant conflict of interest to disclose.

## Authors’ contributions

KN conceived and designed the study. PB, TD, LB, TT, BK, AM, AB, SS, GO and MW assessed the patient and acquired clinical data. PB and KN analysed and interpreted the data. MW and AB performed work on food flours/powders. RH performed serological analyses. PB, and KN drafted the manuscript. All authors revised the manuscript and approved the final version.

## Supplementary Material

Additional file 1: Table S1 Omalizumab dosing according to weight and total IgE levels. **Table S2.** Food combinations. Click here for file
