# Supplementary material for: Phase 1 results of safety and tolerability in a rush oral immunotherapy protocol to multiple foods using Omalizumab
Source: Allergy Asthma Clin Immunol. 2014 Feb 20;10(1):7. doi: 10.1186/1710-1492-10-7 (PMC3936817; doi:10.1186/1710-1492-10-7)
Supplement: Additional file 1: Table S1 — Omalizumab dosing according to weight and total IgE levels. Table S2. Food combinations. [file 1710-1492-10-7-S1.docx]

**SUPPLEMENTAL TABLES**

**Table E1 – DBPCFC schedule**

| Dose in mg of protein | Dosing interval in minutes |
| --- | --- |
| 0.1 | 15 |
| 1.6 | 30 |
| 6 | 45 |
| 25 | 60 |
| 50 | 60 |
| 100 | 120 |

**Table E2- Omalizumab dosing according to weight and total IgE levels**

| Dosing  Interval | Screening  IgE (IU/mL) | Body weight  (kg)/ Study drug dose  (mg) |  |  |  |  |  |  |  |  |
| --- | --- | --- | --- | --- | --- | --- | --- | --- | --- | --- |
| Every 4  weeks |  | **15-20 kg** | **21-25 kg** | **26-30 kg** | **31-40 kg** | **41-50 kg** | **51-60 kg** | **61-70 kg** | **71-80 kg** | **81-90 kg** |
|  | **30-100** | 75 mg | 75mg | 75mg | 75mg | 150mg | 150mg | 150mg | 150mg | 150mg |
|  | **101-200** | 150 mg | 150 | 150 | 150 | 300 | 300 | 300 | 300 | 300 |
|  | **201-300** | 150 mg | 150 | 150 | 225 | 300 | 300 |  |  |  |
|  | **301-400** | 225 mg | 225 | 225 | 300 |  |  |  |  |  |
|  | **401-500** | 225 mg | 225 | 300 |  |  |  |  |  |  |
|  | **501-600** | 300 mg | 300 | 300 |  |  |  |  |  |  |
|  | **601-700** | 300 mg | 300 mg |  |  |  |  |  |  |  |

| Dosing  Interval | Screening  IgE (IU/mL) | Body  weight  (kg)/ Study drug dose  (mg) |  |  |  |  |  |  |  |  |
| --- | --- | --- | --- | --- | --- | --- | --- | --- | --- | --- |
| Every 2  weeks |  | **15-20 kg** | **21-25 kg** | **26-30 kg** | **31-40 kg** | **41-50 kg** | **51-60 kg** | **61-70 kg** | **71-80 kg** | **81-90 kg** |
|  | 201-300 |  |  |  |  |  |  | 225mg | 225mg | 225mg |
|  | 301-400 |  |  |  |  | 225mg | 225 | 225 | 300 | 300 |
|  | 401-500 |  |  |  | 225mg | 225 | 300 | 300 | 375 | 375 |
|  | 501-600 |  |  |  | 225mg | 300 | 300 | 375 | 450 | 525 |
|  | 601-700 |  |  | 225mg | 225 | 300 | 375 | 450 | 525 | 600 |
|  | 701-800 | 225mg | 225mg | 225 | 300 | 375 | 450 | 450 | 600 | * |
|  | 801-900 | 225mg | 225 | 225 | 300 | 375 | 450 | 525 | * | * |
|  | 901-1000 | 225mg | 225 | 300 | 375 | 450 | 525 | 600 | * | * |
|  | 1001-1100 | 225mg | 225 | 300 | 375 | 450 | 600 | * | * | * |
|  | 1101-1200 | 300mg | 300 | 300 | 450 | 525 | 600 | * | * | * |
|  | 1201-3000 | 300mg | 300 | 375 | 450 | 525 | * | * | * | * |
|  | 1301-1500 | 300mg | 300 | 375 | 525 | 600 | * | * | * | * |

**TABLE E3 – FOOD COMBINATIONS IN RUSH mOIT GROUP**

| **Number of foods dosed** | **Number of subjects** | **Food combination** |
| --- | --- | --- |
| 5 | 1 | Peanut, Egg, Pecan, Cashew, Almond |
| 5 | 1 | Peanut, Milk, Walnut, Pecan, Cashew |
| 5 | 2 | Peanut, Walnut, Pecan, Cashew, Hazelnut |
| 5 | 1 | Peanut, Milk, Egg, Cashew, Almond |
| 5 | 1 | Peanut, Milk, Egg, Walnut, Almond |
| 5 | 1 | Peanut, Milk, Egg, Pecan, Cashew |
| 4 | 1 | Peanut, Milk, Egg, Cashew |
| 4 | 1 | Peanut, Walnut, Pecan, Cashew |
| 4 | 1 | Peanut, Milk, Egg, Almond |
| 4 | 1 | Peanut, Milk, Walnut, Cashew |
| 4 | 1 | Peanut, Milk, Egg, Wheat |
| 4 | 1 | Peanut, Milk, Egg, Almond |
| 4 | 1 | Peanut, Cashew, Walnut, Sesame seed |
| 3 | 1 | Peanut, Egg, Cashew |
| 3 | 1 | Walnut, Pecan, Hazelnut |
| 3 | 1 | Walnut, Pecan, Cashew |
| 3 | 1 | Walnut, Cashew, Almond |
| 2 | 2 | Milk, Egg |
| 2 | 2 | Egg, Wheat |
| 2 | 2 | Peanut, Cashew |
| 2 | 1 | Cashew, hazelnut |
